# Cognitive remediation therapy for patients with anorexia nervosa: preliminary findings

**DOI:** 10.1186/1744-859X-6-14

**Published:** 2007-06-05

**Authors:** Kate Tchanturia, Helen Davies, Iain C Campbell

**Affiliations:** 1 Section of Eating Disorders, Institute of Psychiatry, King's College London, London, SE5 8AF, UK

## Abstract

**Background:**

Anorexia nervosa (AN) is a severe mental illness. Drug treatments are not effective and there is no established first choice psychological treatment for adults with AN. Neuropsychological studies have shown that patients with AN have difficulties in cognitive flexibility: these laboratory based findings have been used to develop a clinical intervention based on Cognitive Remediation Therapy (CRT) which aims to use cognitive exercises to strengthen thinking skills.

**Aims:**

1) To conduct a preliminary investigation of CRT in patients with AN 2) to explore whether cognitive training improves performance in set shifting tasks 3) to explore whether CRT exercises are appropriate and acceptable to AN patients 4) to use the data to improve a CRT module for AN patients.

**Methods:**

Intervention was comprised of ten 45 minute sessions of CRT. Four patients with AN were assessed before and after the ten sessions using five set shifting tests and clinical assessments. At the end, each patient wrote a letter providing feedback on the intervention.

**Results:**

Post intervention, three of the five set shifting assessments showed a moderate to large effect size in performance and two showed a large effect size in performance, both indicative of improved flexibility. Patients were aware of an improvement in their cognitive flexibility qualitative feedback was generally positive towards CRT.

**Discussion:**

This preliminary study suggests that CRT changed performance on flexibility tasks and may be beneficial for acute, treatment resistant patients with AN. Feedback gathered from this small case series has enabled modification of the intervention for a future larger study, for example, by linking exercises with real life behavioural tasks and including exercises that encourage global thinking.

**Conclusion:**

This exploratory study has produced encouraging data supporting the use of CRT in patients with AN: it has also provided insight into how the module should be tailored to maximise its effectiveness for people with acute AN.

## Background

Anorexia Nervosa (AN) is a serious mental disorder with a prevalence rate of about 1% and a standardized mortality rate of about 10% [1]. Treatment is problematic and Steinhausen [2] reviewing 119 studies concluded that AN still has a relatively poor prognosis. Chronicity of illness and obsessive personality symptoms are unfavourable prognostic characteristics [3]. The NICE guidelines [4] have concluded that there is currently no recommended psychological treatment nor is there substantial evidence supporting pharmacological interventions [5,6].

Empirical studies have reported that people with AN have difficulties in set shifting tasks, meaning that they find it hard to switch from strategy to strategy, from one stimulus to another and to multitask [7,8]. Such cognitive inflexibility is the prevalent thinking style in AN patients and simply gaining weight does not improve cognitive performance [9-11]. Set-shifting difficulties have been observed in laboratory settings but also has face validity as patients have been consistently described clinically as having persistent, rigid, conforming and obsessional behaviours [12,13]. Thinking style can, therefore, be considered to be a core component to the pathology of AN, maintaining cycles of AN as well as being an obstacle to patients benefiting and completing more emotionally driven psychological treatments [14].

Although there is neuropsychological data showing that people with AN have problems with basic thinking skills, neuropsychological processes and thinking skills are not addressed in current treatments [14]. In the treatment of other psychiatric disorders, for example, schizophrenia, neuropsychological processes and thinking skills are being addressed and it has been demonstrated that cognitive remediation therapy (CRT) improves working memory, planning skills and flexibility [15]. It is hypothesised that CRT works by 1) training basic brain processes via the proliferation and refining of neural connections and 2) teaching adaptive strategies. Thus, the primary function of CRT is to improve the thinking ***process ***rather than the ***content***. In people with AN, an important strategy is the targeting and improving of set-shifting skills.

The purpose of this small case series was to explore: 1) whether therapeutically addressing thinking style improves performance in neurocognitive tasks (primary outcomes) 2) if this intervention is an acceptable treatment for AN patients and 3) how patient and therapist's feedback from a case series can help tailor exercises for inclusion in a manualised intervention package.

For this small case series of patients with AN, a battery of exercises was taken from the flexibility module used as part of the remediation therapy for schizophrenia and adapted and expanded to form the core of the intervention.

## Methods

### Participants

Four patients signed up for the intervention from the South London and Maudsley NHS Trust (SLAM) Eating Disorders Service. Ethics approval was obtained from SLAM and the Institute of Psychiatry Ethics committee. Patients were informed as to the purpose of the treatment and that they could withdraw at any stage.

**Figure 1:**
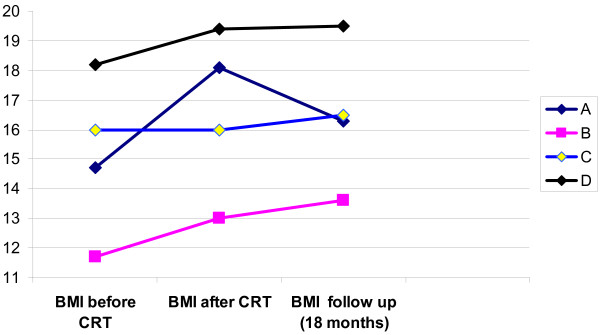
**BMI of patients – before and after CRT intervention and at 18 month follow up**. Age of the patients and duration of illness were as follows: [A – 21 (7); B – 42 (24); C-27 (10); D-22 (7)]; BMI = weight in kilograms/height^2^.

All of the patients were female, diagnosed cases of AN [Body Mass Index (BMI) <17.5] and had received treatment as usual as specified by the inpatient Maudsley Model. Patients' ages were between 21 and 42. Duration of illness was between 7–24 years and age of onset was between 14–18 years (Figure 1). The number of previous admissions ranged from 1 to 3. As CRT aims to target chronically ill patients, these four cases met this criteria.

### Assessments before and after the intervention

Neuropsychological assessments were conducted with each participant before and after the intervention. These assessments tested various aspects of cognitive flexibility and included:

#### The cat bat task [16]

Participants are asked to fill in missing letters in a written short story as quickly and accurately as possible. In the first part of the story, the contextual requirements prompt the participant filling in the letter 'c' and reconstructing the fragment word as 'cat'. In the second part of the story (the shifting part), the word 'cat' is no longer appropriate and the context requires to fill in the letter 'b' and reconstruct the word as 'bat'. Thus, in the first part, participants are primed for the reconstruction of one word (cat) and in the second part they need to adjust their cognitive set to the contextual changes. Perseverative errors and the time taken to complete the task are measured.

#### The trail making task [17]

A computerised version was used in which the task is presented on a VDU and a mouse is used for responding. There are three levels: a motor control task in which responses are made to a shifting 'ball', an ascending alphabetic sequence and an alphabetic and numeric sequence. Cognitive set – shifting is measured by this task.

#### The Brixton test [18]

The participant is asked to predict the movements of a blue circle, which changes location after each response. A concept (rule) has to be inferred from its movements to make correct predictions. Occasionally, the pattern of movement changes and the participant has to abandon their old inferences. Cognitive set-shifting is measured by this task.

#### The haptic illusion task

[19] is a perceptual set-shifting task. This version uses three wooden balls: two small balls of equal size (5 cm dia) and one larger ball (8 cm dia). Participants are asked to judge the relative size of two balls in their hands while keeping their eyes closed. First, the larger ball and one of the smaller balls are placed into participant's hands. This process is repeated 15 times (the same ball is placed in the same hand each time). Then, during the 'critical' stage (30 presentations), participants are given the two identical 5 cm balls, one in each hand. They are asked if there is any difference in size between the balls. Most healthy control participants have the illusion that the ball in the hand previously holding the larger ball is smaller. The number of trials where illusions are experienced is a measure of perceptual rigidity.

#### Self report

To determine levels of obsessionality, the Maudsley Obsessive-Compulsive Inventory [MOCI] [20], a self-report 30 item instrument was completed by the patients. The total score is the sum of the item scores. The self-report Hospital Anxiety and Depression Scale (HADS) [21] was used to measure current anxiety and depression.

### The intervention

The set shifting module was based on the schizophrenia cognitive remediation model originally designed by Delahunty and Morice [22]. Cognitive task selection for the AN module was based on research literature on cognitive performance and clinical observations of AN patients' difficulties in cognitive and behavioural domains. The tasks which were included in the AN module were: geometric figures, (a selection of complex geometric shapes are given to the patient to select and describe one for the therapist to draw); illusions, (visual illusion material is used (ie face/vase illusion) to encourage patients to explore the multiple illusions within one picture); Stroop material (to practice switching between attending to different aspects of a stimulus eg colour or word) Manipulations (eg reversing a sequence of letters and finding different permutations for sequences of letters), Infinity Signs (eg drawing figures based on different rules), Line Bisection (marking points on different length lines to encourage estimating), Token Towers (shape sorting task), Hand Tasks (switching between different sequences of hand movements), Maps (finding alternate and quickest routes on a map). All tasks were done using pencil and paper and are given to the patient with instructions from the therapist. A monitoring form was used to report patient performance (scoring 1–3 poor/good) and exercises were timed. The patient was asked to generally reflect on the tasks in terms of thinking style. Each patient received 10 sessions of CRT each lasting approximately 45 minutes. The therapist used a motivational non-judgemental approach.

## Results

### Quantitative data

Main clinical characteristics before CRT and immediately after are presented together with BMI, levels of depression and anxiety and as obsessive compulsive characteristics (Table 1).

**Table 1:** Results from pre and post intervention: clinical characteristic questionnaires for each participant and BMI

	**BMI**		**HADS**	Anxiety	**HADS**	Depression	**MOCI**	
	**Pre**	Post	**Pre**	Post	**Pre**	Post	**Pre**	Post

A	14.70	**18.10**	15.00	15.00	14.00	16.00	10.00	13.00
B	11.70	**13.02**	13.00	**9.00**	9.00	**6.00**	6.00	6.00
C	16.00	16.00	11.00	11.00	4.00	**1.00**	15.00	**12.00**
D	18.20	**19.40**	13.00	**12.00**	5.00	**4.00**	14.00	**8.00**

Changes in measures are presented in bold.

To explore cognitive changes after the intervention, the case series of 4 patients (using effect size Cohen d) was used and compared to published data [16]. Retrospective controls (AN group N = 22) were assessed before and after treatment as usual when the nutritional programme was successful: baseline (BMI = 13.3 – indicating severe underweight condition; outcome 18.4 above diagnostic threshold).

In Table 2, results from the present case series are compared to the effect sizes of 22 patients from a previous cohort who were receiving treatment as usual, but no CRT. As can been seen, the effect sizes from the previous study (ie treatment as usual) are small, meaning that with weight gain alone, neuropsychological performance on shifting tasks has not changed. However, in the present case series of patients receiving CRT as well as treatment as usual, there are medium to very large effect sizes in set shifting performance.

**Table 2:** Set Shifting before and after intervention and effect sizes of cognitive changes

	BT(T1)	BT(T2)	P(T1)	P(T2)	B(T1)	B(T2)	Trt(T1)	Trt(T2)	TRP(T1)	TRP(T2)	I(T1)	I(T2)
A	23.93	30.00	1.00	**.00**	16.00	**13.00**	39.9	**29.0**	1.00	**.00**	12.00	12.00
B	32.83	**23.42**	2.00	**1.00**	16.00	**13.00**	38.7	41.6	.00	.00	16.00	**12.00**
C	25.62	**20.00**	1.00	**.00**	11.00	**5.00**	missing	missing	missing	miss	16.00	**13.00**
D	18.64	**15.06**	.00	**.00**	19.00	**13.00**	18.5	88.0	.00	.00	30.00	**15.00**
M (SD) N = 4	25.2 (5.8)	22.1 (6.2)	1.0 (0.8)	0.2 (0.5)	15.5 (3.3)	11.0 (4.0)	32.4 (11.9)	52.9 (31.0)	0.3 (0.5)	0 0	18.5 (7.8)	13.0 (1.4)
**Effect size**	Medium 0.6		**Large 1.38**		**Large 1.14**		Large 1.1		Large 0.9	**Large 1.1**		
M(SD) Tchanturia et al (2004) Retrospective control (N = 22)	29.0 (13.7)	26(12.4)	1.5 (1.6)	1.0 (1.5)	17.9(9.7)	16.1(6.3)	44.2 (24.3)	44.1(20.0)	1.8(3.3)	2.6(4.5)	13.0(10.7)	10.8 (9.7)
**Effect size**	Small 0.2		Small 0.3		Small 0.2		Small 0.2		Small 0.2	Small 0.2		

Key:

BT – Bat time one (CATBAT story bat time), P – Perseverations in catbat story, B – Brixton number of errors.

Trt – Trail making shifting time, TRP – Trail making perseveration, I-Illusions.

T1 – first assessment, T2 – follow up after 10 sessions of CRT (first four cases) or treatment as usual in inpatient programme.

### Qualitative data

At the end of the ninth session, patients and therapists wrote letters reflecting on the treatment. These were exchanged in the last session and provided an opportunity to explore how acceptable this intervention was for patients.

An aspect of the intervention that seemed appealing for patients was that the exercises and reflection on them involved thought *processes *and not thought *content*. In the patients' letters, CRT is depicted as being useful as a pre treatment, because it does not involve issues relating to emotions, feelings, and content of thought, This is reflected in their letters:

"*It was refreshing to be involved in something that did not focus on emotions and which was entirely separate from the anorexia and related issues" C. "It was so nice that there was no connection to the Eating Disorder and that I was able to concentrate on other aspects of me" C. "CBT and other psychological therapies can be too intense both physically and psychologically at a low weight to be of any benefit" D*. "*Improvement in feeling able to achieve the tasks*" A.

Patients also commented on how the intervention helped with flexibility in both the short and long term "*I found the sessions incredibly helpful as I find being flexible very difficult" D. "The short term benefits are increasing the ability to be more flexible in set shifting, ie the odd/evens and number manipulation task" A. "The long term benefits still being enforced 6 months on from leaving the ward are an improvement of being able to multi task, therefore enabling quicker and more flexible decision making in everyday life" B. "My thinking seems to have become broader and more creative" B*.

A need for translation of skills into everyday life also became apparent from comments in the letters such as "*The first few sessions gave me time to settle and familiarise myself with the work and also gave me space to explore the possibilities how this could help me. I found that later, after about 4 or 5 sessions, I was finding links between the game playing and how I could be more flexible at home and work" A*.

"*I would have liked more advice on how I could use the principles in my daily life" C. and "towards the end of the last sessions it would be useful to think about how I can use what I have learnt in what I do in my own time" D*.

#### Follow-up

Eighteen months after receiving CRT, each patient in the case series was contacted to obtain follow up information. Our main interests were: 1) BMI, 2) whether they had been re-admitted to the inpatient ward and 3) whether they were using skills and strategies obtained from the CRT sessions.

All patients had maintained a stable BMI [Fig 1] (although lower than the normal range 20–25). None of them had been re-admitted to hospital, and all of them were working or studying.

## Discussion

Our aim was to explore whether a CRT module was acceptable to AN patients, secondly, to establish whether cognitive exercises changed set shifting task performance and finally, based on our results, to modify the CRT manual for a larger pilot study.

As far as the neuropsychological performance was concerned, the observed medium to large effect sizes suggests that targeted cognitive flexibility exercises change performance in shifting tasks on follow up assessment. Comparison with the retrospective data obtained from patients in the same clinical setting and using the same neuropsychological tasks with a treatment as usual group, shows small effect sizes in set shifting performance. It is not possible to draw firm conclusions given the small size of this case series compared against the larger retrospective comparison group.

Based on a) the practical application of the tasks, b) retrospective observations of the cases on supervision and c) qualitative analysis feedback letters, we have established that the treatment package is acceptable. For example, none of the patients dropped out, all commented on the relevance of the exercises and gave useful recommendations for improvements. Therapists reported that the intervention was sufficiently gentle to allow acutely ill patients to access it and further commented that the simplicity and structure of the sessions were helpful in establishing a good relationship with the patient.

One of our aims was to develop and tailor exercises from established interventions and adapt them to produce a CRT intervention for AN patients. This was done in a number of ways from adding new tasks to adjusting the delivery of the intervention. For example, a monitoring form was used to report patient performance (scoring 1–3 poor/good) and exercises were timed. However, this was found to be ineffective without a sufficient baseline and therefore it is proposed that future monitoring of sessions should be done qualitatively by asking the patient questions throughout the session and recording their answers. These will include "What did you learn from these tasks?", "What do the tasks show you about your thinking style?" These questions should allow the patient to internalise the strategy they have used as well as reflect on the tasks in terms of thinking style. The evaluation questions should also provide the therapist with a better insight into the patients thinking style and hence direction on how to proceed in the specific task and also in the sessions.

It was also proposed, based on qualitative feedback by patients (see results), that the therapist should encourage the patient to make connections between thinking styles apparent whilst doing the tasks to real life scenarios. To this end it is proposed that the therapist ask the patient after each task "How does your thinking style [in the task] relate to real life?" As well as making these connections, behavioural tasks that can be undertaken outside of the sessions can be introduced in later sessions to intensify the learning experience. These tasks can be discussed in the session and then carried out by the patient in their own time. Feedback can then be given to the therapist in the following session. A list of behavioural tasks will be included in the updated manual. From the four patients we learned about the possible behaviours patients could try successfully. A few examples of these are reading a newspaper in a different order, taking a different route to proposed destination, using a different mobile phone ring-tone, changing their night time routine, cleaning their teeth with their non dominant hand and, making-up a headline from a newspaper article. Patients were able to carry out such tasks, and it gave them a sense of achievement and intensified the learning experience gained in the laboratory setting.

Therapists' observations and patients' comments have also helped us to improve the module by including extra exercises related to set shifting eg switching attention and embedded words whereby a patient reads through a paragraph of text switching between words relating to 'hot' and 'cold' topics. Other switching tasks that have been added include pictures of objects with an incongruent word written on top and pictures of clock faces where it is required to switch between a 12 hr and a 24 hr clock.

One other way in which the case series has lent to further development of a tailored module comes from the task entitled geometric figures. Therapists found that all four patients found this task quite problematic, because when dictating how to draw the geometric shape, all patients provided unnecessary details and this made interpretation of drawing the figure difficult. This clinical observation is in accord with research evidence that has shown that people with AN pay extensive attention to detail [23-26]. This poor organizational strategy may lead to difficulties in seeing the overall context. In AN, this strategy is not only present in relation to food, but also to other aspects of life, such as work and homework. To help remediate this thinking style and improve global thinking, the revised manual will include two additional tasks to the geometric figures. For example, a task which requires big pieces of written information such as a letter to be made into a headline or a text message and secondly, a task which requires thinking about prioritising information.

## Summary

This current case series has demonstrated that 1) patients enjoyed and completed the CRT intervention 2) performance on cognitive tasks improved and 3) the module could be improved and tailored for people with AN based on feedback from patients and therapists.

It is hard to draw firm conclusions based on four case reports, however, this preliminary exploration shows the following:

This treatment was positively received by patients with a long history of AN and who had several attempts with psychological interventions which may have failed. Patients commented that they found the intervention positive because it was not related to food or emotional material, and the tasks were achievable and fun. Furthermore, patients in this case series found it interesting to explore their thinking strategies and ways of processing information and had a sense of achievement in applying small strategic changes to real behaviours.

Patients' qualitative feedback allowed us to revisit some of the exercises and change instructions and procedures related to the tasks as well as adding more exercises to promote global thinking. We have established that patients with AN are able to reflect effectively on their thinking style from session 3–4 and to start testing out their skills obtained in the sessions in real life situations.

A larger pilot study will allow us to address and explore experiences from this case series and utilise these in a tailored manual for AN patients.
